# Unavailability of experimental 3D structural data on protein folding dynamics and necessity for a new generation of structure prediction methods in this context

**Published:** 2025-07-10

**Authors:** Aydin Wells, Khalique Newaz, Jennifer Morones, Jianlin Cheng, Tijana Milenković

**Affiliations:** 1 Department of Computer Science and Engineering, University of Notre Dame, USA; 2 Institute for Computational Systems Biology, University of Hamburg, Germany; 3 Department of Electrical Engineering and Computer Science, University of Missouri, Columbia, USA

## Abstract

**Motivation:**

Protein folding is a dynamic process during which a protein’s amino acid sequence undergoes a series of 3-dimensional (3D) conformational changes en route to reaching a native 3D structure; the resulting 3D structural conformations are called folding intermediates. While data on native 3D structures are abundant, data on 3D structures of non-native intermediates remain sparse, due to limitations of current technologies for experimental determination of 3D structures. Yet, analyzing folding intermediates is crucial for understanding folding dynamics and misfolding-related diseases. Hence, we search the literature for available (experimentally and computationally obtained) 3D structural data on folding intermediates, organizing the data in a centralized resource. Additionally, we assess whether existing methods, designed for predicting native structures, can also be utilized to predict structures of non-native intermediates.

**Results:**

Our literature search reveals six studies that provide 3D structural data on folding intermediates (two for post-translational and four for co-translational folding), each focused on a single protein, with 2–4 intermediates. Our assessment shows that an established method for predicting native structures, AlphaFold2, does not perform well for non-native intermediates in the context of co-translational folding; a recent study on post-translational folding concluded the same for even more existing methods. Yet, we identify in the literature recent pioneering methods designed explicitly to predict 3D structures of folding intermediates by incorporating intrinsic biophysical characteristics of folding dynamics, which show promise. This study assesses the current landscape and future directions of the field of 3D structural analysis of protein folding dynamics.

## 1 Introduction

Protein folding is a process by which a protein’s amino acid sequence folds into a three-dimensional (3D) structure (or conformation). The 3D structure directs what other biomolecules the protein may interact with to carry out its function(s) [3, 17]. When a protein fails to fold correctly, its misfolded 3D structure can disrupt normal cellular functions and contribute to diseases [22, 53]. Protein folding is a dynamic process, as it involves a protein’s sequence gradually folding into a series of 3D structural conformations until arriving at a final, native structure of the protein [50, 58]; this time series of 3D structural changes (including the native state) is called a *protein folding pathway*. The dynamics of the folding process can be studied from two prominent perspectives: (i) *post-translational* folding and (ii) *co-translational* folding.

**Figure 1:**
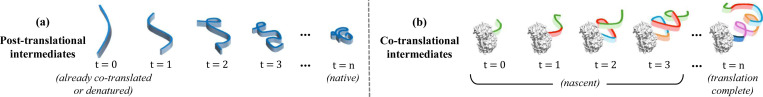
Illustrations of **(a)** post-translational and **(b)** co-translational protein folding pathway intermediates. **(a)** A protein’s *entire* sequence (blue line) undergoes conformational changes. The resulting 3D structures from time 0 to time *n* are the *n* + 1 intermediates in the post-translational pathway. **(b)** A protein’s nascent sequence undergoes conformational changes as newly translated amino acids are added (as indicated by the additional line color) by the ribosome (the grey entity) at time *t* = *k* to its already translated part from the previous time step (*t* = *k* − 1), until all amino acids have been translated (*t* = *n*). The resulting 3D structures are the *n* + 1 intermediates in the co-translational pathway. In both panels, the first *n* intermediates (from time 0 to time *n* − 1) are non-native, and the last intermediate (at time *n*) is native.

Post-translational folding (also referred to as folding in vitro [8] or in solution [58]) is concerned with the structural changes of a protein’s *entire* sequence, which may have already gone through co-translational folding (defined below) or instead may start from a denatured state [5]. Specifically, a post-translational protein folding pathway is a time-series of 3D conformational changes that a protein’s full sequence undergoes until reaching a native state (Fig. 1(a)) [5, 10]. The 3D conformations formed during this process (including the native structure) are called post-translational protein folding pathway intermediates (or just post-translational intermediates, for simplicity) [49].

Co-translational folding is concerned with the structural changes of a protein’s *partial* sequence as it gradually grows *during* its translation by the ribosome. Namely, a co-translational protein folding pathway is a time-series of conformational changes that a protein’s sequence undergoes as newly translated amino acids are added to its already translated part from the previous time step, until all amino acids have been translated (Fig. 1(b)) [37, 44, 58]. The 3D conformations corresponding to the gradually increasing subsequences formed during translation (including the native structure) are called co-translational protein folding pathway intermediates (or just co-translational intermediates) [25].

Note that both a post-translational and co-translational non-native intermediate can be classified as *on-pathway*, meaning one that is trajected to reach the native state, or *off-pathway*, meaning one that represents a misfolded state that must be resolved before correct folding can continue [4, 7].

Analyzing folding intermediates is crucial for understanding protein folding dynamics and deepening insights on protein functions and misfolding-related diseases. For example, the rate of protein folding dynamics can help explain folding efficiency, i.e. how quickly a protein becomes functional [52, 57]. So, information on intermediates corresponding to slow vs. fast folding could help explain why folding rate differences lead to differences in folding efficiency [54].

Some data types – specifically kinetics and thermodynamics data – do exist on a somewhat large scale that aim to capture information on folding dynamics. For post-translational folding, databases with kinetics (protein folding rate) data exist for dozens to hundreds of proteins. For example, the Protein Folding DataBase (PFDB) contains kinetics (folding rate constant) data for 141 (89 two-state and 52 multi-state) single-domain globular proteins; a two-state protein folds via a two-state mechanism, directly from unfolded to folded, and a multi-state protein folds via a multi-state mechanism involving more than two intermediates. As of its publication in 2019, this made PFDB the largest available database of protein folding kinetics [31]. As another example, Start2Fold contains kinetics (hydrogen/deuterium exchange) data extracted from the literature for 57 proteins with 219 residue sets (i.e. conformations), where each protein may have several residue sets categorized based on so-called protection levels (i.e. early, intermediate, and late for folding; and strong, medium, and weak for stability) [48]. For post-translational folding, thermodynamics data also exist on a large scale. For example, ProThermDB contains thermodynamics (e.g. melting temperature and free energy) data for 31,580 (12,050 wild-type and 19,530 mutated) proteins [43]. For co-translational folding, we could not identify any organized database containing either kinetics or thermodynamics data. Instead, we could identify some isolated studies that provide data of these types for a handful of proteins (Supplementary Section S1.1).

Protein folding kinetics and thermodynamics data are valuable for understanding folding dynamics [15, 64]. However, both provide quantitative measures of the protein folding process, and typically, a quantity per protein rather than per intermediate of a protein. In other words, they lack 3D spatial resolution at the level of intermediates to reveal the atomic-level interactions and structural transitions that underpin the protein folding process. To understand folding dynamics more deeply, it is desirable to also study 3D structures of folding intermediates. So, a goal of this paper is to provide a centralized resource discussing current data of this type, which as we will show later is quite sparse. We comment on the availability of 3D structural data on folding intermediates obtained (i) experimentally and (ii) computationally.

First, biophysical technologies such as NMR, cryo-EM, and X-ray crystallography [27] have contributed a wealth of experimentally determined 3D structural data that are publicly available in the Protein Data Bank (PDB) [59]. These data are almost exclusively for native states of proteins rather than for non-native folding intermediates. This is because NMR and cryo-EM may capture instances of intermediate states, but their resolution or sensitivity may fall short when trying to detect a rapidly fluctuating sequence of conformational changes [27]; and X-ray crystallography requires proteins to form crystals, a challenge that folding intermediates often cannot meet due to their unstable and dynamic nature [27, 65]. Moreover, the protein folding process (the folding of secondary and tertiary structures rather than necessarily the complete protein synthesis) often occurs in microseconds to seconds [8, 33], making it difficult for the traditional technologies to achieve the temporal resolution necessary to capture 3D structures of folding intermediates in real time [14, 30].

Second, the availability of large-scale experimentally derived data on native 3D structures has facilitated the development of computational approaches for predicting native 3D structures on an even larger scale [1, 46, 61]. As we will show, there is a lack of large-scale experimentally determined 3D structural data on non-native folding intermediates. Given this, an interesting question arises: can computational approaches for native 3D structure prediction be used to predict 3D structures of non-native folding intermediates? For post-translational intermediates, this question was already asked recently, revealing a lack of success when using eight prominent approaches of this type, including AlphaFold2 [46]. More recently, a computational approach was introduced specifically for predicting 3D structures of post-translational intermediates; its predictions aligned relatively well with experimental observations on protein folding mechanics (rather than with 3D structural data) for a handful of proteins [24]. However, this attempt focused on *post-translational* intermediates. Our literature search done as a part of this study has found no attempts focused on predicting 3D structures of *co-translational* intermediates; an exception is a study posted as a pre-print after the completion of all of our analyses and close to the completion of our paper, which covers one protein [58]. Hence, as another contribution of this study, we introduce an original research evaluation of whether AlphaFold2 can correctly predict experimentally determined 3D structures of co-translational intermediates that are currently available for a handful of proteins.

**Figure 2:**
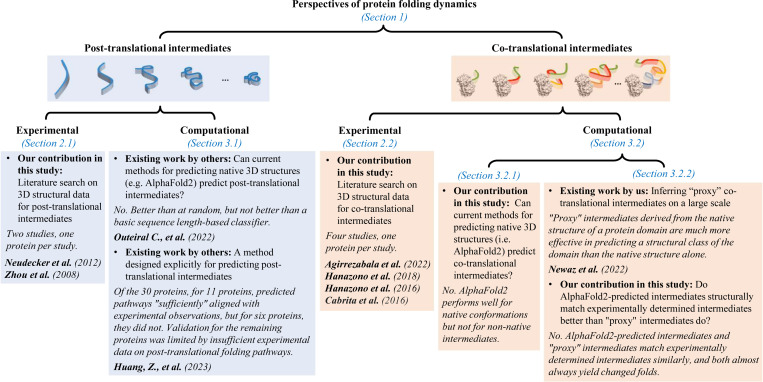
Summary of this paper that is focused on availability and analysis of 3D structural data related to protein folding dynamics.

In this paper (Fig. 2), we perform a thorough literature search for currently available experimentally determined (Section 2) and computational (Section 3) 3D structural data on folding intermediates, both post-translational (Sections 2.1 and 3.1) and co-translational (Sections 2.2 and 3.2) ones. Also, we investigate whether current native 3D structure predictors can correctly predict 3D structures of post-translational intermediates (from existing literature; Section 3.1) as well as co-translational intermediates (from our own analysis original to this paper; Section 3.2). We aim to inform the community about available 3D structural data on folding intermediates and a (potential) need for a new generation of protein 3D structure predictors in this context.

Note that other families of computational approaches for studying protein folding dynamics exist, namely molecular dynamics simulations, Monte Carlo simulations, and coarse-grained models [28]. These are not a focus of our paper. Yet, we will touch on them again in Section 3.1.

## 2 Experimental 3D structural data on protein folding pathway intermediates

### 2.1 Experimental post-translational intermediates

Our literature search on experimentally determined 3D structural data for post-translational intermediates identifies two studies [38, 66] that explicitly documented 3D structures of intermediates in PDB (Supplementary Fig. S1 and Supplementary Section S1.2). Each of the two studies investigated one protein. The two proteins have sequence lengths of on the order of 100 amino acids. Each study reported two post-translational intermediates per protein – one pre-native and one native. For each intermediate, a deposited sequence range (i.e. the amino acid sequence that is reported) and a modeled sequence range (i.e. the portion of the sequence that has available 3D structure information) were reported in PDB; note that the two can differ.

While these two studies have successfully resolved 3D structures of intermediates, they are limited in scope – the number of proteins studied (only two), the size of proteins studied (quite short), and the number of intermediates reported (only two). As the current 3D structural data on post-translational intermediates is sparse, owing to the limitations of biophysical technological discussed above, there is a need for novel technologies for this purpose.

For each study’s protein, we examine the 3D structural similarity between the given protein’s first intermediate and its second intermediate, in terms of the Template Modeling score (TM-score) [62]. TM-scores range between 0 and 1, where scores below 0.17 indicate random-like similarities, scores above 0.3 indicate significant similarities, and scores above 0.5 indicate the same overall fold (Supplementary Section S1.3). We find that the TM-score between the two intermediates is reasonably high, between 0.77 − 0.80, depending on the protein (Supplementary Fig. S1). Also, for completeness, we examine each study’s aim in more detail; due to space constraints, we report this in Supplementary Section S1.2.

### 2.2 Experimental co-translational intermediates

**Figure 3:**
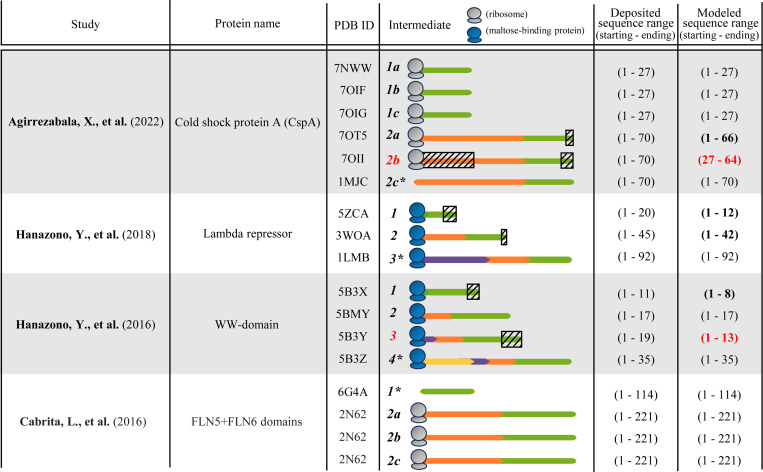
Details of the four studies we have identified that report 3D structural data of co-translational intermediates. The protein from the first study listed has intermediates for two deposited sequence ranges (1: 1–27, 2: 1–70). For each of the two intermediates, three distinct conformations are provided (a, b, and c). Conformation 2c is the native structure of the protein (as denoted by an asterisk); see Supplementary Section S1.5. Similar holds for the last study listed, whose protein has intermediates corresponding to two sequence ranges (1: 1–114, 2: 1–221). The former intermediate is the native structure (Supplementary Section S1.5), and for the latter intermediate, three distinct confirmations are provided (a, b, and c; note that 3D structures of all three of these are available in the same PDB file, with the same PDB ID 2N62). In the second and third studies listed, the given protein has three (1–3) and four (1–4) intermediates, respectively, of growing length, each with a single conformation. For both studies, the last intermediate is the native structure. The third column contains the PDB ID of each conformation of an intermediate. In the fourth column, each conformation is a colored line. The same color (e.g. green) in different lines for the same protein/study indicates the same subsequence of the considered protein whose structure may have changed due to the existence of other subsequences (e.g. orange, purple, yellow). Different colors (e.g. green vs. orange) in the same line indicate different subsequences of the considered protein that are translated at different time steps; subsequences that are translated earlier are towards the right in the line (N-terminus), and those that are translated later are towards the left in the line (C-terminus). For example, for the protein from Hanazono et al. (2018) [21], intermediate 1 spans amino acids 1–20 (green - newly translated), intermediate 2 spans amino acids 1–45 (1–20: green – the same amino acids as in the first intermediate, 21–45: orange – newly translated amino acids), and intermediate 3 spans amino acids 1–92 (1–20: green – the same amino acids as in the first intermediate, 21–45: orange – the same amino acids as in the orange portion of the second intermediate, 46–92: purple – newly translated amino acids). One of our goals is to then measure 3D structural changes between the green portions of intermediates 1, 2, and 3, and between the green+orange portions of intermediates 2 and 3, in order to quantify the level of change due to co-translation. Note that in the fourth column, the gray or blue entity at the C-terminus is a ribosome or maltose-binding protein, respectively; only the conformations with PDB IDs 1MJC and 6G4A do not have such an entity attached to them because these intermediates were experimentally studied in the absence of either entity. The fifth and sixth columns are defined in the same way as in Supplementary Fig. S1. A modeled sequence range is bolded if a portion of the deposited sequence is missing from the modeled sequence; such a discrepant subsequence is represented with a hatched box overlaid on the deposited sequence in the fourth column (Supplementary Section S1.3 discusses how we handle 3D structural comparison in Section 3.2 and later in the presence of minor subsequence discrepancies). A modeled sequence range is in red if its corresponding conformation is excluded from subsequent analyses in Section 3.2 and later because of major subsequence discrepancies considered to affect data completeness/quality (Supplementary Section S1.6).

Our literature search on experimentally determined 3D structural data for co-translational intermediates identifies only four studies that explicitly document 3D structures of intermediates in PDB: Agirrezabala et al. (2022) [2], Hanazono et al. (2018) [21], Hanazono et al. (2016) [20], and Cabrita et al. (2016) [6] (Fig. 3 and Supplementary Section S1.4). The current data on co-translational intermediates suffer from similar limitations as the data on post-translational intermediates – each of the four studies on co-translational intermediates also reports a single protein, the studied proteins are also relatively short (all but one of the four studies analyze proteins with fewer than 100 residues), all four studies also provide quite few (2–4) intermediates. So, the current data on co-translational intermediates are also sparse. This further emphasizes the need for innovative experimental biophysical technologies for better capturing data on intermediates.

Across the four studies/proteins, there are 11 distinct intermediates. Just as multiple 3D structural conformations may be reported for a native state, multiple conformations may be reported for an intermediate state as well. For three out of the 11 intermediates, three conformations have been reported per intermediate; for the remaining eight intermediates, only one conformation has been reported per intermediate (Fig. 3). Hence, there are a total of 17 conformations for the 11 intermediates. For six of the conformations, their modeled sequences are shorter than their deposited sequences (PDB IDs 70T5, 70II, 5B3X, 5B3Y, 5ZCA, 3W0A). Of the six, we discard from further analyses the conformations with PDB IDs 70II and 5B3Y, and we continue to analyze the remaining four conformations; Supplementary Section S1.6. Hence, we proceed with analyzing a total of 15 conformations for 10 intermediates (i.e. all but the two red ones in Fig. 3).

We measure structural similarities (TM-scores) between all pairs of (partial and full) conformations of intermediates of the same protein (Supplementary Section S1.3). We find the following (Supplementary Fig. S2). First, we focus on distinct conformations for the same intermediate (e.g., 2a vs. 2b vs. 2c from a given study). All corresponding TM-scores are below 0.5, indicating that no two conformations of the same intermediate have the same fold. Second, we focus on the conformational change of a sequence of an intermediate gradually over time (e.g., a green sequence in an intermediate vs. the same green sequence in the next intermediate for the same protein). Overall, we observe quite a large conformational change of the same sequence between consecutive time points in the presence of additional amino acids being translated and added to the 3D structure, often resulting in a changed fold (i.e. TM-score below 0.5) of the given sequence during translation. Third, to evaluate the effect of time passed, we compare conformations corresponding to the same sequence of an intermediate at closer vs. more distant times (e.g., a sequence from time 1 to time 2 vs. the same sequence from time 1 to time 3). In all comparisons, more time-distant conformations of intermediates tend to show higher levels of conformational change than time-closer conformations for the same protein. For more detailed results, see Supplementary Section S1.7.

For completeness, we examine aims of the four studies from this section in more detail. Due to space constraints, we report this in Supplementary Section S1.4.

## 3 Computational 3D structural data on protein folding pathway intermediates

### 3.1 Computational post-translational intermediates

With advancements in protein 3D structure prediction, available data on native structures has increased drastically. For example, prior to the release of the AlphaFold database, ∼200,000 experimentally determined 3D structures were reported in PDB – just a fraction of the billions of known protein sequences [55]. Now, computationally predicted 3D structures are available for over 214 million proteins [55]. Given the lack of experimentally determined 3D structural data on folding intermediates, can existing methods for predicting native 3D structures accurately predict 3D structures of intermediates?

Outeiral et al. (2022) investigated this question for eight prominent existing methods, including AlphaFold2, on *post-translational* intermediates [46]. They compiled a dataset of 170 proteins from PFDB [31] and Start2Fold [48]. Each protein was assigned one of two folding kinetics classes – two-state or multi-state. 90 out of the 170 proteins were two-state, and the remaining 80 were multi-state. Additionally, 79 out of the 90 two-state proteins had associated folding kinetics data (i.e. folding rate constants).

To predict a protein’s folding pathway, Outeiral et al. modified implementations for seven of the considered methods for predicting native 3D structures to also output 3D structures produced during the process of predicting the native structure. For the remaining method – AlphaFold2 – predicted pathways were received from DeepMind. Regarding the number of pathways per protein: For each of the 170 proteins, each of the seven methods (except AlphaFold2) was used to predict 10 pathways per protein. Five of the more scalable methods were also used to predict 200 pathways per protein. For AlphaFold2, only one predicted pathway was available per protein. Regarding the number of intermediates per pathway, we could not find this information in the paper by Outeiral et al. Also, we could not find the predicted structures of the intermediates reported in that paper.

When evaluating a method’s predicted pathway, Outeiral et al. simply assumed that many proteins fold by first forming secondary structures, followed by forming tertiary contacts between those secondary structures. So, Outeiral et al. examined a predicted pathway using its predicted 3D structural intermediates to track when native contacts (contacts found in the native structure) formed between each pair of secondary structure elements over time. If all pairs formed their native contacts around the same time, this indicated a two-state folding mechanism, where folding occurs in a single concerted step. In contrast, if some pairs formed contacts earlier and others later, this suggested the presence of intermediate states, characteristic of a multi-state folding mechanism. Because folding pathways for a protein varied across different prediction runs, some proteins showed both two-state and multi-state behavior. Thus, Outeiral et al. defined the fraction of two-state pathways as the probability that a protein exhibited two-state folding kinetics. This information was then used to evaluate whether a method’s predicted pathways (i) are predictive of a protein’s folding kinetics class (i.e. two-state or multi-state), and (ii) correlate with experimentally measured folding rate constants.

For the first evaluation, all structure prediction methods achieved statistically significant yet quite modest performance. The methods were compared to a trivial baseline relying only on chain length. This simple baseline outperformed all structure prediction methods. For the second evaluation, chain length had the strongest (and correctly-signed) correlation, again outperforming any structure prediction method. For more detailed results, see Supplementary Section S1.8.

These findings suggested that folding pathways predicted by the existing structure prediction methods fail to meaningfully capture folding kinetics data or correlate with experimental folding rate data. In other words, the existing methods fail to accurately model post-translational folding pathways, which calls into question their ability to provide meaningful insights into folding dynamics. Thus, the Outeiral et al. study emphasizes the need for a new generation of computational approaches that can accurately predict 3D structures of intermediates on post-translational protein folding pathways.

The existing methods were originally designed to predict native 3D structures, and they were applied by Outeiral et al. (2022) in a naïve way to generate pathways that yielded the native structure, without any direct capability to capture protein folding dynamics. On the other hand, Pathfinder is a recent method by Huang et al. [24] designed specifically to predict a post-translational folding pathway, i.e. to more explicitly capture the dynamics of post-translational protein folding. Pathfinder predicts a protein’s post-translational pathway via three steps: (i) it identifies seed states representing key intermediate conformations along a protein’s folding pathway; (ii) it computes transition probabilities between seed states to map out all the plausible folding pathways a protein might take; and (iii) it identifies a path through the seed states that maximizes the overall transition likelihood (for details, see Supplementary Section S1.9). This results in a predicted folding pathway consisting of a small number of key seed states, representing the most probable sequence of structural transitions. The seed states included in this “optimal” pathway are designated as a given protein’s predicted post-translational pathway intermediates.

Pathfinder was applied to 30 proteins (Supplementary Section S1.10). To assess whether a protein’s predicted post-translational pathway captured realistic folding dynamics “sufficiently” well, Huang et al. compared structural features of the predicted pathway to known experimental folding behavior of the given protein. Specifically, Huang et al. computed the contact order (i.e. average sequence distance between residues that form native contacts) in each intermediate of the predicted pathway. Then, by *qualitatively* analyzing how contact order evolved over the course of the predicted pathway and comparing this information to experimentally observed folding data, Huang et al. evaluated whether a protein’s predicted pathway “sufficiently” captured realistic folding dynamics (“sufficiently” because the evaluation was qualitative and thus likely subjective, based on descriptive folding information from the literature, rather than quantitative and thus objective).

Pathfinder predicted post-translational folding pathways “sufficiently” well for 11 out of the 30 proteins, while it did not predict them well for six other proteins. For the remaining 13 proteins, although relevant literature describing their folding pathways existed (which is why these proteins were chosen in the first place), Huang et al. noted that their predicted pathways needed “further validation because of the lack of intermediate state data” in the literature. Despite this, Huang et al. observed that in proteins containing both *α*-helices and *β*-strands, the earliest intermediate states often featured *α*-helical structures. Given that *α*-helices are thought to be generally more stable, this observation aligned with existing experimental findings suggesting that *α*-helices tend to form earlier in the folding process. Huang et al. viewed this as an encouraging sign that Pathfinder captured biologically meaningful folding behavior, even in cases where more detailed validation was currently lacking.

Huang et al.’s results highlighted both the promise and current limitations of Pathfinder’s approach – while it can “sufficiently” capture realistic folding mechanisms for a subset of the 30 proteins studied, its broader applicability is constrained by the lack of availability of folding dynamics information on post-translational protein folding pathways.

Pathfinder is a Monte Carlo approach for predicting post-translational folding pathways, using probabilistic sampling to explore conformational space and infer likely folding pathways [24]. This method complements other common computational strategies such as molecular dynamics simulations (which provide atomistic detail through force field-driven trajectories) and coarse-grained models (which simplify molecular representations to enable broader exploration of folding behavior) [28]. Despite their differences, all three approach types – Monte Carlo, molecular dynamics simulations, and coarse-grained – share limitations, specifically a reliance on highly parameterized energy functions, simplified assumptions (e.g. modeling only backbone or only side-chain atoms, or grouping several atoms into a single unit), or constrained timescales, which can introduce significant biases in predictions of protein folding dynamics and a limited simulated timescale [16, 23, 28]. Moreover, these approaches (including those used in the Outeiral et al. study and Huang et al.’s Pathfinder) rely on experimental observations of secondary structure formation or folding kinetics information for validation – rather than direct comparison to 3D structures of intermediates (as such data do not exist for post-translational intermediates other than the two studies mentioned in Section 2.1). We imagine that having 3D structural data on post-translational intermediates could help better evaluate and refine folding pathway prediction tools.

### 3.2 Computational co-translational intermediates

#### 3.2.1 Can current methods for predicting native 3D structures predict co-translational intermediates?

Despite significant advances in protein structure prediction, our literature search has uncovered no computational methods for predicting 3D structures of co-translational intermediates – with the sole exception of a very recent pre-print, released after the completion of our analyses and near the completion of our paper, which addresses a single protein [58]. So, as an original contribution to this paper, we explore whether AlphaFold2, a prominent existing method designed for prediction of native 3D structures, can predict 3D structures of co-translational intermediates – following a similar question that Outeiral et al. (2022) [46] investigated for post-translational intermediates. While the studies from Section 3.1 evaluated predicted post-translational intermediates with respect to quantitative measures (e.g. folding kinetics data) or qualitative information (e.g. secondary-structure formation information), we cannot do the same for predicted co-translational intermediates, because such data are limited for co-translational intermediates (Section 1). Instead, we predict 3D structures of co-translational intermediates for the four proteins that have experimentally determined 3D structures of co-translational intermediates available (Section 2.2). Then, we evaluate the predictions by directly comparing them to the 3D structures of experimentally determined co-translational intermediates. In this analysis, we rely on the same 15 conformations for 10 intermediates as in Supplementary Fig. S2, i.e. all but the two red ones in Fig. 3, which we have already cleaned to remove data noise (Supplementary Section S1.6), hence hopefully yielding higher-confidence results from our analyses. We predict 3D structures of the co-translational intermediates as follows.

For each of the 15 conformations of the intermediates, we give its deposited sequence (excluding the ribosome or maltose-binding protein) as input into AlphaFold2 (ColabFold [34] v1.5.2). As a result, each conformation has a corresponding AlphaFold2-predicted 3D structure. AlphaFold2 generates five predicted structures for each sequence, ranked by confidence. We report results for the top-ranked predicted structure. Nonetheless, results for all five AlphaFold2-predicted structures of a given sequence are qualitatively similar and quantitatively almost identical regardless of which AlphaFold2-predicted structure for a given sequence is used (Supplementary Fig. S3). Note that we use AlphaFold2 rather than AlphaFold3 because the main improvements of the latter compared to the former focus on modeling protein complexes and biomolecular interactions, rather than offering tremendously significant advances for predicting the native structure of a single-chain protein [1, 9].

**Figure 4:**
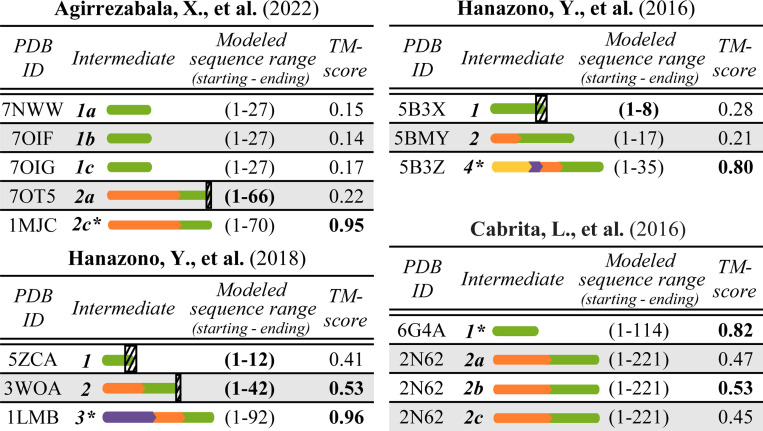
Structural similarities in terms of TM-scores between the AlphaFold2-predicted vs. experimentally determined structures for the same 15 conformations of the 10 co-translational intermediates as in Supplementary Fig. S2, i.e. for all but the two red ones in Fig. 3. For each of the four studies, there is a corresponding table; the first three table columns are already explained in Fig. 3 and Supplementary Fig. S2 (except that here we do not show or analyze an attached entity at the C-terminus). The fourth table column reports the TM-score between an AlphaFold2’s prediction and the corresponding experimentally determined confirmation; all TM-scores above 0.50 are bolded, indicating the same overall fold. 3D visualizations of the AlphaFold2-predicted structures are shown in Supplementary Fig. S4. Note that each result here is for the highest-ranked AlphaFold2-predicted structure; results for all five highest-ranked AlphaFold2-predicted structures are shown in Supplementary Fig. S3.

To evaluate how well AlphaFold2 predicts 3D structures of co-translational intermediates, for each conformation, we compare its experimentally determined structure to its AlphaFold2-predicted structure using TM-score (Supplementary Section S1.3). Our findings are as follows (Fig. 4). Unsurprisingly, AlphaFold2 performs well for native conformations of intermediates (TM-scores in the 0.80–0.96 range, depending on the protein). There is a stark contrast in its performance for non-native intermediates, where it does not work as well. Namely, of the 15 conformations of the intermediates that are evaluated, 11 are non-native. For nine of these, AlphaFold2 yields TM-scores below 0.5, specifically in the 0.14–0.47 range (indicating different folds), and for the remaining two non-native conformations, AlphaFold2 yields TM-scores of just above 0.5. Due to space constraints, for key observations per study, see Supplementary Section S1.11.

Our findings reinforce that current computational structure prediction methods (like AlphaFold2) that are designed to predict native 3D structures are not inherently able to capture well co-translational intermediates. This observation is consistent with the previous findings for post-translational intermediates [46] (Section 3.1).

#### 3.2.2 Inferring “proxy” co-translational intermediates on a large scale

Although computational analyses of *post-translational* folding have been done on at least somewhat large scale for dozens to hundreds of proteins – though with non-3D structural data (Section 3.1) – for *co-translational* folding, we could only analyze four proteins for which 3D structures of their intermediates are available, since no quantitative nor qualitative data exist on co-translational intermediates (Section 3.2.1). This raises the question of whether 3D structures of co-translational intermediates can be predicted on a larger scale, and if so, how they would be validated. A promising recent method used atomistic simulations to predict 3D structures of co-translational intermediates by capturing both native and non-native interactions [58]. However, this method was applied to and evaluated on only one protein. Moreover, our analysis in Section 3.2.1 reveals that one of the best existing methods designed to predict native 3D structures – AlphaFold2 – is not equipped to capture structures of co-translational intermediates. Certainly, testing current and future methods designed for predicting 3D structures of post-translational intermediates on predicting 3D structures of co-translational intermediates could be a valuable future direction, although validation would be challenging given the lack of data on co-translational intermediates. So, how may it be possible to predict and validate co-translational intermediates on a much larger scale? In our recent work, we took a step forward in this direction, by utilizing a bulk of data that is readily available – native 3D structures – to infer “proxy” co-translational intermediates on a large scale [41].

Specifically, we extracted a protein’s 3D structural “proxy” co-translational intermediates from its native structure as follows. Motivated by the intuition that during co-translation of a protein, newly translated amino acids are gradually added to its already translated parts from the previous time steps (Fig. 1b), the protein’s first “proxy” intermediate contains the first *k* amino acids of the entire sequence (and the corresponding substructure of the native structure), the second “proxy” intermediate contains the first 2*k* amino acids of the entire sequence (and the corresponding substructure of the native structure), the third “proxy” intermediate contains the first 3*k* amino acids of the entire sequence (and the corresponding substructure of the native structure), etc; this continues until arriving at the last “proxy” intermediate, which captures the entire protein’s sequence (and the entire native 3D structure) [41]. Note that in our original study, we used *k* = 5 because this intuitively mimics the addition of individual 3D secondary structural elements (i.e., *α*-turns or *β*-strands) [41]. As a contribution to this study, we evaluate the effect of alternative values for *k*, confirming *k* = 5 to be the most meaningful choice (Supplementary Fig. S5).

**Figure 5:**
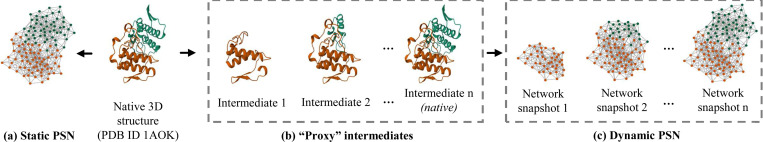
Illustration of **(a)** a static protein structure network (PSN), **(b)** “proxy” co-translational intermediates, and **(c)** dynamic PSN, all for the same protein with PDB ID 1AOK. The static PSN captures the native structure of the protein. “Proxy” co-translational intermediates capture gradually increasing portion of the protein sequence and the corresponding portion of the native 3D structure, with the last “proxy” intermediate capturing the entire sequence and native structure. Each “proxy” intermediate (intermediate 1, 2, …, n) corresponds to a network snapshot (network snapshot 1, 2, …, n, respectively) in the dynamic PSN. The figure has been adapted from Newaz et al. (2022) [41].

The set of all “proxy” intermediates for a protein aims to mimic the process of the protein undergoing co-translational folding (Fig. 5b). We say “mimic”, because unfortunately this procedure is unable to capture any conformational changes of an intermediate over time, i.e. any actual dynamics of the co-translational folding process, because all of the intermediates are extracted from the same, static native structure. Although these intermediates do not explicitly model co-translational folding as it occurs in vivo (hence the term “proxy”), they were the most practical large-scale method for mimicking co-translational folding, i.e. the best one could do on a large scale at the moment without significant computational and methodological innovation.

We evaluated our approach for extracting “proxy” structural intermediates in the context of protein structure classification (PSC) [29] – a task that is closely related to protein function prediction [18]. PSC is a supervised problem of assigning proteins into pre-defined structural classes based on the proteins’ features [39]. Traditional 3D-structural features are extracted directly from 3D structures. Instead, one can first model a 3D structure as a protein structure network (PSN), where nodes are amino acids and two nodes are joined by an edge when the corresponding amino acids are close enough in the 3D structure [11]. By converting a 3D structure into a PSN, a wealth of network-based methods can be used to extract network features for use in the PSC task (which otherwise could not be extracted directly from 3D protein structures).

In a previous study, our lab demonstrated that using PSN features extracted from native structures often proved to be more accurate and typically faster than using sequence and non-network-based 3D structural approaches in the PSC task [39]. However, traditional PSN approaches (including our lab’s previous approaches) modeled a native 3D structure as a *static* PSN (Fig. 5a), disregarding the dynamic process of co-translational folding. This is because experimental data on dynamics (i.e. on co-translational intermediates) are lacking (Section 2.2). However, with the availability of “proxy” co-translational intermediates, we were able to model a native 3D structure as a *dynamic* (Fig. 5c) rather than static PSN [41]. The desire to model a native 3D structure as a dynamic PSN, in order to capture, even implicitly, the dynamics of protein folding, was the reason why we came up with the idea of “proxy” co-translational intermediates.

We hypothesized that modeling a 3D structure as a dynamic PSN would be more informative than modeling it as a static PSN, in the task of PSC. Both network types (dynamic vs. static PSNs) were used to model the exact same data. So, any improvements in PSC accuracy when using dynamic PSNs compared to using static PSNs would be a direct result of including the dynamic information into the network model.

We tested this hypothesis on ∼44,000 protein domains for which both 3D structures and structural class information were available. Each domain was modeled as a dynamic PSN (*k* = 5) as well as a static PSN. To fairly compare dynamic and static PSNs, we extracted comparable network features from the two PSN types, namely dynamic and static graphlets, respectively; graphlets are Lego-like building blocks of a network [40]. These features were used within a logistic regression classifier. Dynamic and static PSNs were evaluated in the task of classifying the ∼44,000 protein domains with respect to their structural classes. Dynamic PSNs statistically significantly (corrected p-value of below 10^−6^) outperformed their static counterpart. Also, dynamic PSNs showed a low misclassification rate (i.e. the fraction of PSNs for which an incorrect structural class was predicted) of up to 10% in ∼89% of all evaluation tests. These results confirmed that considering dynamic information, even only as “proxy” co-translational intermediates derived from native structures, could yield better insights into folding-related phenomena (membership in structural classes) than static PSNs.

The PSC task is not explicitly designed to address protein folding *dynamics*. So, as an original contribution to this study, we evaluate “proxy” intermediates in a different task more directly related to folding dynamics. Specifically, we assess 3D structural similarity (as measured by TM-score) between experimentally determined intermediates and their corresponding “proxy” intermediates. We do not anticipate a perfect structural match between the two. This is because the former often undergo conformational changes during co-translation (as the TM-scores from Supplementary Fig. S2 show), but this is not reflected in the design of the latter (as the “proxy” intermediates are extracted from a native structure). So, the comparison between experimentally determined intermediates and “proxy” intermediates is not our key goal here. Instead, it is to evaluate whether the experimentally determined intermediate conformations are structurally matched better or worse by their corresponding “proxy” intermediates or by their corresponding AlphaFold2-predicted intermediates. If better, this would further highlight AlphaFold2’s limitations in modeling protein folding dynamics and suggest that “proxy” intermediates may offer a more meaningful (yet quite imperfect, due to being derived from native structures) representation of folding dynamics.

When we analyze a subset of the four studies/proteins with available experimentally determined 3D structures of co-translational intermediates that meet relevant criteria (Supplementary Section S1.12), our findings are as follows (Supplementary Table S1). The AlphaFold2-predicted intermediates match the experimentally derived intermediates just as poorly as the “proxy” intermediates, which are simply derived from native structures. All TM-scores are quite low, between 0.21 − 0.53. AlphaFold2 performs better (yet not too well, as indicated by the TM-scores) for a half of the analyzed data, while “proxy” intermediates perform better (also not too well) for the other half.

We have shown in our previous study [41] that using “proxy” intermediates in a dynamic fashion – to the best of our knowledge, the only feasible large-scale 3D structural option of the dynamic nature – is a powerful method for capturing more information from native 3D structures in the task of PSC than analyzing the native structures in a traditional, static fashion. In the above analysis, we have shown that “proxy” intermediates perform comparably to AlphaFold2 in the task more closely related to protein folding dynamics – predicting 3D structures of co-translational intermediates. Yet, we do not aim to imply that “proxy” intermediates are an appropriate approach for modeling 3D structures of folding intermediates, and thus for capturing folding dynamics. Instead, our findings imply that AlphaFold2 does not consistently outperform the baseline “proxy” intermediates in the second task, and it also most often yields TM-scores below 0.5 that indicate a changed fold, all of which underscores the need for new types of approaches that will be capable of accurately modeling folding dynamics and predicting 3D structures of intermediates.

## 4 Discussion and concluding remarks

Our study (and others) highlights a fundamental barrier to advancing the understanding of protein folding dynamics: the near-total absence of 3D structural data on folding intermediates, in the context of both post-translational and co-translational folding. Our literature search has revealed only two studies that report experimentally determined 3D structures of post-translational intermediates, and only four studies that report such data for co-translational intermediates. Each of these proteins is constrained by a limited number of intermediates, and most by short length.

While thermodynamic and kinetic datasets do exist on a somewhat broader scale for post-translational folding, they typically provide a single quantity per protein rather than per intermediate along a folding pathway. Even in the best cases, where some proteins have a measurement per intermediate, the data are only quantitative, lacking 3D spatial information. For co-translational folding, the situation is even more sparse; there are no centralized resources containing data of these types; instead, only a few isolated studies provide such data, typically with one protein per study. As a result, while thermodynamic and kinetic data allow for quantitative analyses of post-translational folding, their lack of accompanying 3D structural information constrains the depth of such analyses. For co-translational folding, the near-complete absence of both quantitative and 3D structural data for intermediates leaves little means for any analysis.

Given the lack of experimentally determined 3D structural data on intermediates, it is natural to ask whether computational methods – especially those that have demonstrated success in predicting native 3D structures – can help bridge the gap. Our findings from Section 3.2, along with those of Outeiral et al. (2022)[46] discussed in Section 3.1, provide a strong caution against this assumption. These findings reinforce the conclusion that existing structural prediction methods do not generalize well outside of native conformations, and thus cannot be expected to accurately predict 3D structures of intermediates along a folding pathway.

So, how may this be achieved accurately? One possibility may be to retrain existing structure prediction methods with data on folding intermediates, and in doing so, re-engineer these approaches to predict the 3D structures of folding intermediates. However, this direction is limited by the near-total lack of experimentally determined 3D structural data on intermediates, both for post-translational and co-translational folding. Another possibility may be to develop methods that more accurately account for the underlying biochemical and physical factors driving protein folding dynamics, such as the geometry of the ribosome (and its tunnel), presence of chaperones, or vectorial, time-dependent nature of translation (in the case of co-translational folding) [2, 44, 58]. Also, incorporating codon usage patterns might improve the prediction of co-translational folding pathways because it serves as a proxy for translation rates, which are evolutionarily tuned to regulate the timing of protein synthesis [47]. For instance, slower translation rates allow the N-terminal portion of a protein to fold independently before the synthesis of the C-terminal portion is complete, while faster translation rates can help prevent the buildup of certain folding intermediates [37]. Even synonymous codon usage – where different codons code for the same amino acid – can affect translation speed and, as a result, impact co-translational folding [37, 42].

Recent efforts have begun to demonstrate the potential of considering these factors for better prediction of folding pathways. For post-translational folding, Huang et al. (2023) [24] did so via Pathfinder (Section 3.1). For co-translational folding, we and others have modeled the vectorial nature of protein synthesis by explicitly capturing increasingly longer subsequences of a protein and then predicting their 3D substructures as the structures of co-translational intermediates; the latter has been achieved in three distinct ways, as follows.

First, we previously introduced “proxy” intermediates [41] – 3D substructures representing progressively longer C-terminal sequence fragments of a protein extracted *naïvely from its native structure* – to model co-translational folding; while “proxy” intermediates do not capture protein folding dynamics *explicitly*, we showed that their analysis is nonetheless significantly more accurate than traditional analyses of only the native structures in the task of predicting protein structural classes (Section 3.2.2). Second, we have used a more advanced idea of predicting 3D structures of co-translational intermediates by feeding progressively longer C-terminal sequence fragments of a protein into AlphaFold2; however, this approach was unable to accurately predict the existing experimentally determined co-translational intermediates (Section 3.2.1), likely because it does not effectively learn the kinetics (and thus dynamics) of protein folding [46]. Third, a different existing structure prediction method that predicts protein folding pathways while accounting for *non-native* interactions and that account for some key principles of co-translational folding – was used on increasingly longer subsequences of a protein to predict their 3D structures. Namely, the study by Wang et al. (2025) [58] – which we again note appeared as a pre-print *after* the completion of all of our analyses – used Monte Carlo simulations that “recapitulate intrinsic properties of co-translational folding, such as the sequential emergence of the peptide and its propensity to form secondary and tertiary structures” and also “provide insights into the thermodynamic stability and dimensions of folding intermediates, which determine whether structural elements can form inside the narrow ribosome exit tunnel” [58]. To evaluate the predicted 3D structures resulting from these simulations, Wang et al. (2025) performed in vitro experiments using truncated protein domains (sequence fragments of increasing length) attached to a ribosome that mimicked different stages of co-translation. The experimentally measured structural compactness and stability of these domains were then compared to the corresponding simulation-predicted conformations and stability profiles, and the two seemed to align.

When a new method becomes available for predicting the 3D structures of folding intermediates, an immediate challenge will be the evaluation of its predictions, i.e., assessment of whether the predicted 3D structural intermediates match biological reality. This can be approached in several ways.

First, ideally, evaluation would involve 3D structural comparison between predicted and experimentally determined folding pathways. Although such experimental data are scarce, they are not entirely absent; such data exist for post-translational as well as co-translational folding for a handful of proteins (Section 2); this is similar to the initial scarcity of available protein 3D structures at the inception of PDB (see below and Supplementary Fig. S6(a)). Given a predicted pathway and an experimentally determined pathway, one strategy to perform their 3D structural comparison could be to generalize established structural similarity measures from comparing two native structures to comparing corresponding pairs of intermediates along the two pathways. This is precisely the strategy we have used to evaluate AlphaFold2-predicted co-translational intermediates in Section 3.2.1. While we have used TM-score (for reasons discussed in Supplementary Section S1.3), additional 3D structural similarity measures can also be used, which are sometimes in agreement with and sometimes complementary to each other, depending on whether they are global vs. local, superposition-based vs. superposition-free, or considering all atoms vs. only selected subsets of atoms [45]. Another quite natural strategy for 3D structural comparison of the two pathways could rely on dynamic PSNs. Namely, PSNs have been powerful in tasks such as protein structural classification [11, 39] as well as protein function prediction [18]. And of all PSN types, dynamic PSNs have shown the greatest accuracy [41]. Dynamic PSNs were introduced exactly for the purpose of modeling 3D structures of intermediates along a co-translational pathway (Section 3.2.2). So, each of a predicted pathway and an experimentally determined pathway can naturally be converted into its respective dynamic PSN. Then, the two pathways, i.e., their dynamic PSNs, can be compared using a wealth of approaches for dynamic network comparison [56, 67]. This is especially true given the rapid advancement of artificial intelligence, including deep learning on graphs [18, 67], as well as geometric deep learning combined with network analyses [36, 35]; these kinds of approaches have already shown strong performance in a range of structural biology tasks such as drug binding, protein–protein interaction prediction, and fold classification [67].

Second, when experimentally determined 3D structures of intermediates along a pathway are unavailable, one evaluation strategy could be to use quantitative data (e.g., folding rate constants or thermodynamic stability), which are more widely available than 3D structural data, especially for post-translational intermediates, like what Outeiral et al. (2022) [46] and Huang et al. (2023) [24] did (Section 3.1). An alternative evaluation strategy could involve performing in-house wet-lab experimental validation, as done by Wang et al. (2025) [58], per our discussion earlier in this section.

In summary, the problem of protein folding dynamics is challenging, including due to data sparsity. However, such type of challenge is not without precedent. At the inception of PDB [59] in 1976, only 13 structures were available. For its first decade, structural growth was slow, with only 6–32 additional structures being added to PDB each year (Supplementary Fig. S6(a)). Yet over time, PDB has grown to include over 200,000 structures (Supplementary Fig. S6(a)). In parallel, the launch of the Critical Assessment of protein Structure Prediction (CASP) competition [61] in 1994, with 27 teams and 186 predictive methods (Supplementary Fig. S6(b)), sparked rapid progress in native structure prediction, culminating in tools like AlphaFold2 and an explosion of predicted 3D structures – with over 214 million in the AlphaFold Protein Structure Database alone [55]. This history suggests that major progress can begin with limited data. Such a growing trajectory, likely with a much faster timeline due to developments in artificial intelligence and machine learning, may be possible for data on 3D structures of folding intermediates if the community begins to systematically organize existing and generate new 3D structural data, however sparse; this has been exactly a key goal of our paper. Like the early PDB and CASP efforts on native structures, such a foundation could catalyze innovation in intermediate-specific experimental technologies as well as computational prediction methods (importantly, including evaluation frameworks).

Addressing the current impasse will require coordinated, closely intertwined efforts across wet lab experimental and computational communities. On the experimental side, new technologies are needed to capture transient 3D structures of intermediates with higher resolution and faster acquisition times. On the computational side, more efforts are needed to develop intermediate-specific prediction methods informed by curated experimental 3D structural data as well as kinetics or thermodynamics data. Additionally, we encourage the computational community to include analyses of computational complexity as well as results on running times and memory usage when evaluating new methods, as such key data are often overlooked in the current literature. Developing accurate and efficient computational approaches is only part of the endeavor; ensuring these methods are user-friendly and broadly accessible is equally critical for their widespread adoption. This includes offering intuitive interfaces and well-documented software, providing open-source implementations, and ensuring compatibility with platforms and workflows commonly used by the community.

## Supplementary Material

Supplement 1

## Data Availability

Only existing, already published data and software are used in this paper, all of which are publicly available and appropriately referenced throughout the paper.
