# Supplementary material for: Unavailability of experimental 3D structural data on protein folding dynamics and necessity for a new generation of structure prediction methods in this context
Source: ArXiv. 2025 Jul 10:arXiv:2507.08188v1. Preprint. [Version 1] (PMC12265586)
Supplement: Supplement 1 [file NIHPP2507.08188v1-supplement-1.pdf]

Supplementary Information

Section S1 Supporting text for the main paper

Section S1.1 Additional details on kinetics and thermodynamics data related to co-translational folding

Here, we complement Section 1 in the main paper by discussing existing kinetics and thermodynamics data related to co-translational folding.

For co-translational folding, we could not identify any organized database containing kinetics or thermodynamics data. Instead, we could identify some isolated studies that provide data of these types for a handful of proteins, as follows. Samelson et al. [51] reported kinetics (co-translational folding rate) data for a protein called HaloTag, Farias et al. [12] studied the thermodynamic stability of the S6 protein, and Kelkar et al. [26] captured kinetics data for intermediates of the protein mCherry at specific time points.

Section S1.2 Additional information about the studies on experimentally determined 3D structures of post-translational intermediates

Here we complement Section 2.1 in the main paper by providing more details about the two studies on post-translational intermediates:

Neudecker et al. (2012) [38] addressed the question of how folding intermediates contribute to amyloid fibril formation, a key process in many neurodegenerative disorders. While such intermediates have been implicated in aggregation, the structural mechanisms underlying this transition remain poorly understood. To investigate this, Neudecker et al. used NMR spectroscopy to determine the structure of a post-translational intermediate of the Fyn SH3 domain. Neudecker et al. found that the intermediate exhibited a disordered C-terminus, exposing an aggregation-prone β-strand. Two pathway intermediates are provided in the study: the first intermediate is an "early-stage" structure (PDB ID: 2L2P) and the second intermediate is the native structure (PDB ID: 2L25) of the SH3 domain.

Zhou et al. (2008) [66] explored the folding mechanisms of ribonuclease H (RNase H) in order to enhance understanding of the principles governing protein folding dynamics. Utilizing multidimensional NMR, Zhou et al. identified a compact intermediate of RNase H that formed rapidly during folding, exhibiting a native-like core of helices. The study's findings supported a hierarchical folding mechanism, where secondary structures formed first, followed by tertiary contacts. Additionally, Zhou et al. findings indicated that the intermediate was well-defined and folded into its native structure more quickly because of this intermediate. Two post-translational intermediates are provided in the study: the first intermediate is an "early-stage" structure (PDB ID: 2RPI) and the second intermediate is the native structure (PDB ID: 1RIL) of the RNase H protein.

Section S1.3 Additional details about our use of TM-score

Here we provide details on our use of TM-score [60, 62].

TM-score is a widely used quantitative measure for assessing the level of similarity between two 3D structures. It is a *global* measure, meaning that it evaluates the overall agreement of entire folds between two 3D structures, even if some of their peripheral regions might differ [62].

In contrast to global 3D structural similarity measures, *local* measures assess structural similarity at a finer spatial scale; these measures typically involve calculating the spatial distance deviations of pairs of residues (typically $C\alpha$ atoms) within a *local neighborhood*, and then aggregating scores over only short-range residue interactions. As such, local structural similarity measures are sensitive to local distortions, making them useful for evaluating the accuracy of smaller structural regions regardless of the global fold [32].

It was recently shown that global measures of 3D structural similarity – including TM-score and Global Distance Test (GDT) – are "extremely highly correlated" [45], which supports the use of TM-score as a representative global measure. The same study found that local measures – including local Distance Difference Test (lDDT), Recall, Precision and F-measure (RPF), and Contact Area Difference (CAD) – are also "highly correlated" with each other [45]. Further, that study found that the local similarity scores are often in agreement with global ones, as well as that the methods for predicting 3D structures (referred to as "models" in that study) that are selected as the best with respect to local measures (including lDDT and RFP) are typically also among the best methods selected according to the global measures (including TM-score and

GDT) [45]. Yet, the two groups of measures, global vs. local, as well as individual measures within each group, often have (dis)advantages [45].

Since one aim of our study is to determine whether two compared 3D structures (as described in cases (i) and (ii) below) have the same *overall* fold, we use TM-score, which is global, as our primary measurement for assessing 3D structural similarity. As mentioned above, local structural similarity measures might provide valuable complementary information in certain aspects, particularly when the global fold is preserved. However, we argue that if the global fold is not preserved between the compared 3D structures – as we have observed in our analyses of non-native folding intermediates, where TM-scores are almost always below 0.5 – identifying specific regions of local deviation would offer limited structural insights, as the underlying folds are fundamentally distinct. Given this, and given the above discussion of frequent agreement/consistency as well (rather than just complementarity) between results of global and local measures, we believe that the use of TM-score as our measure of choice in our study is justified as well as sufficient.

We use the TM-score [62] software available through the Zhang lab's web server (https://zhanggroup.org/TM-score/). We compare 3D structures of two intermediates (i.e., their sequences) in terms of TM-score as follows. We either compare (i) the experimentally determined 3D structure of a given sequence to the experimentally determined 3D structure of the same sequence *at a different time* (Section 2 in the main paper), or (ii) the *experimentally determined vs. predicted* 3D structures a given sequence (Section 3.2 in the main paper). Because there exist some (minor) discrepancies between the deposited and modeled sequences of a given intermediate (the hatched boxes in Fig. 3 in the main paper) for several of the 15 considered conformation of the 10 considered intermediates (all but the two red conformations of intermediates in Fig. 3 in the main paper), there also may exist discrepancies between the modeled sequences of two compared intermediates. If so, we handle these discrepancies when computing TM-scores between two modeled sequences as follows. For case (i) above, we first identify and extract the longest common contiguous subsequence (LCCS) shared between the experimentally determined compared modeled sequences. Each of the two resulting LCCS modeled sequences is then converted into PDB format and uploaded to the TM-score web server mentioned above using the "Structure 1" and "Structure 2" input fields. Then, the web server returns the corresponding TM-score. For case (ii) above, recall that we predict a 3D structure for an intermediate by inputting the *deposited* sequence of the intermediate into AlphaFold2 (Section 3.2.1 in the main paper). Then, when we compare a predicted 3D structure and its experimentally determined counterpart. Here, we first extract the LCCS between the deposited sequence of the predicted 3D structure and the modeled sequence of its corresponding experimentally determined 3D structure. Second, the two resulting 3D structures corresponding to the two LCCSs are then converted into PDB format and uploaded to the TM-score web server mentioned above using the "Structure 1" and "Structure 2" input fields. Then, the web server returns the corresponding TM-score. Note that in either of cases (i) and (ii) above, we do not need to normalize a given TM-score with respect to the length of either of the two input 3D structures, because we compare only the LCCS shared between the pair; i.e., the two structures inputted into the TM-score web server always have the exact same sequence.

To reemphasize, this methodology is applied uniformly across all analyses presented in Sections 2 and 3.2 of the main paper where TM-score is employed.

TM-scores range from 0 to 1, with higher values indicating greater structural similarity between protein 3D structures. In more detail [60]: A TM-score below 0.17 indicates a random-like structural similarity, i.e. that the probability of finding a TM-score this low from randomly chosen structural pairs is close to 1. A TM-score higher than 0.3 indicates a significantly high structural similarity, i.e. that the probability of finding such a score from random structural pairs is very low. A TM-score of 0.5 is generally considered a key threshold, as a TM-score higher than 0.5 suggests that the compared 3D structures have the same overall fold or similar topology according to structural classifications such as CATH [19] and SCOPe [13].

Section S1.4 Additional information about the studies on experimentally determined 3D structures of co-translational intermediates

Here we complement Section 2.2 in the main paper by providing more details about the four studies on co-translational intermediates:

Agirrezabala et al. (2022) [2] investigated how early folding events are influenced by the ribosome – and hence the shape of protein structures – during translation. Specifically, Agirrezabala et al. observed the folding of a β-barrel protein (specifically a cold shock protein (CspA)) and reported its interactions with the ribosome during translation. Using cryo-EM, Agirrezabala et al. captured a co-translational intermediate of CspA that revealed an initial α-helical conformation that formed within the ribosome's exit tunnel before transitioning

into its native β-strand structure upon emergence. The study's findings emphasized the ribosome's role in shaping early folding events and suggested that co-translational folding may be a common feature of β-barrel proteins. Agirrezabala et al. provided the native structure of the CspA protein (PDB ID: 1MJC), three different conformations of the CspA protein when 27 amino acids have already been translated (PDB ID: 7NWW, 7OIF, 7OIG), and two different conformations of the CspA protein when 70 amino acids have already been translated (PDB ID: 7OT5, 7OII).

Hanazono et al. (2018) [21] explored the co-translational folding of nascent polypeptide chains, specifically focusing on the λ repressor N-terminal domain, a small α-helical protein, to uncover the atomic-level details of this process. Using circular dichroism (CD) spectroscopy, Hanazono et al. examined intermediate-length variants of the λ repressor to capture structural intermediates during co-translational folding. The study found that partial helices formed within the ribosome's exit tunnel, with increasing chain length leading to progressive stabilization of secondary and tertiary structure. This suggested a stepwise folding process where local α-helical segments formed early and guided subsequent structural changes. Their results highlighted how the ribosome constrains and influences protein stability and function. Hanazono et al. reported the native structure of the λ repressor (PDB ID: 5ZCA), and the structures of two different co-translational folding intermediates; the first of the two captures the structure of the λ repressor when 20 amino acids have already been translated (PDB ID: 3WOA) and the second captures the structure of the λ repressor when 45 amino acids have already been translated (PDB ID: 1LMB).

Hanazono et al. (2016) [20] investigated the co-translational folding of nascent proteins, which are affected by the rate of translation by the ribosome. While fully translated proteins are capable of achieving their native conformation, nascent protein structures primarily begin folding co-translationally at their N-terminal regions; however, the transient structures of intermediates involved in this process remain poorly understood. Hanazono et al. focused on the early folding events of the WW domain, a small β-sheet protein, during translation. Using CD spectroscopy, N-terminal fragments of the WW domain were examined to determine how partial sequences adopted structure before arriving at the native structure. Hanazono et al. showed that isolated N-terminal fragments lack stable β-sheet formation, suggesting that the WW domain required a sufficiently long polypeptide chain before β-strands can properly fold. This contrasts with α-helical structures, where partial helices formed early. Hanazono et al. findings highlighted a cooperative folding mechanism for β-sheet proteins, where both chain length and long-range molecular interactions played a key role in stabilizing the WW domain's structure. The study reported the native structure of the WW domain (PDB ID: 5B3Z), and the structures of three different N-terminal fragments of the WW domain: the first of the three captures the structure of the WW domain when 11 amino acids have already been translated (PDB ID: 3WOA), the second of the three captures the structure of the WW domain when 17 amino acids have already been translated (PDB ID: 5BMY), and the last of the three captures the structure of the WW domain when 19 amino acids have already been translated (PDB ID: 5B3Y).

Cabrita et al. (2016) [6] addressed the question of how ribosomes influence protein folding to ensure proper structural formation of nascent proteins. Using NMR spectroscopy, Cabrita et al. examined the conformations of a ribosome–nascent chain complex (comprised of an N-terminal domain FLN5 and a C-terminal domain FLN6) during co-translation. By progressively shortening the translated polypeptide chain, the study found that the ribosome modulated folding – FLN5 remained unfolded beyond the tunnel, while isolated FLN5 folded spontaneously, even when truncated. The study's results emphasized the ribosome's active role in shaping protein conformations during co-translation. Cabrita et al. reported the native structure of only the FLN5 domain in isolation, with length of 114 amino acids, (PDB ID: 1QFH – now updated to 6G4A) and the three different conformations of the complex's full structure (i.e. FLN5+FLN6), with length of 221 amino acids (PDB ID: 2N62).

Section S1.5 Additional details about Fig. 3 in the main paper

Here, we provide additional details to the legend of Fig. 3 in the main paper.

For the protein from Agirrezabala et al. (2022) [2], the asterisk next to conformation 2c (PDB ID 1MJC) denotes that this conformation is the native structure of the protein (that is no longer attached to the ribosome, i.e., the grey entity shown in other conformations). Conformations 2a and 2b have the the same deposited sequence range as conformation 2c, but are conformations attached to the ribosome and are observed in the study to have structural differences to conformation 2c due to the appearance of the ribosome.

For the protein from Cabrita et al. (2016) [6], the asterisk next to intermediate 1 (PDB ID 6G4A) denotes that this intermediate is the native structure of the protein (that is no longer attached to the ribosome). Intermediate 1 has been experimentally observed to achieve a native structure without the rest of the

translated sequence (shown in intermediate 2), but in this study structural changes were observed to occur when translation continued past the first intermediate (hence the three conformations of intermediate 2).

Section S1.6 Additional details about discarding particular conformations from subsequent analyses

Here we complement Section 2.2 in the main paper with additional reasoning behind discarding particular conformations.

Recall that for six conformations of the intermediates, their modeled sequences are shorter than their deposited sequences (namely, those with PDB IDs 7OT5, 7OII, 5B3X, 5B3Y, 5ZCA, 3W0A). We discard from further analyses the conformation of an intermediate with PDB ID 7OII due to a huge data loss of ∼50% in the modeled sequence compared to the deposited sequence. Also, we discard from further analyses the intermediate with PDB ID 5B3Y, both because this is the second largest data loss, and because this intermediate's deposited sequence adds only two extra amino acids (deposited sequence range of 1-19) to the intermediate with PDB ID 5BMY (deposited sequence range of 1-17), while at the same time 5B3Y loses many of its amino acids in the modeled sequence but 5BMY does not lose any. Hence, 5BMY is an intermediate of much higher data quality, and again, almost as long as the deposited sequence of 5B3Y. We continue to analyze the remaining four conformations (i.e., 7OT5, 5B3X, 5ZCA, 3W0A) with some minor data loss.

Section S1.7 Results on structural similarities between conformations of intermediates for the same protein, for experimental co-translational folding data

Here we complement Section 2.2 in the main paper by reporting key observations and methodological details about structural similarities in terms of TM-scores between all possible pairs of (partial as well as full) conformations of intermediates of the same protein, for experimental co-translational folding data. Relevant results are shown in Supplementary Fig. S2.

First, we focus on different conformations for the same intermediate, which only the studies by Agirrezabala et al. (2022) and Cabrita et al. (2016) have. In other words, we focus on 1a vs. 1b vs. 1c as well as 2a vs. 2c in the former, and 2a vs. 2b vs. 2c in the latter. All of the corresponding TM-scores are below 0.5, indicating that no two conformations of the same intermediate have the same fold.

Second, we focus on the conformational change of a sequence of an intermediate gradually over time, which all four studies have. For example, we focus on the conformation of a green sequence in one intermediate vs. the conformation of the same green sequence in the next intermediate (for the same protein), or on the conformation of a green+orange sequence in one intermediate vs. the conformation of the same green+orange sequence in the next intermediate (for the same protein). Overall, we observe quite a large conformational change of the same sequence over time in the presence of additional amino acids being translated and added to the 3D structure, most often resulting in a changed fold of the given sequence during translation. In more detail, for the protein from Agirrezabala et al. (2022), we observe the fold change in all comparisons (all TM-scores are 0.33 or lower). For the protein from Hanazono et al. (2018), we also observe the fold change in all comparisons (all TM-scores are 0.43 or lower). For the protein from Hanazono et al. (2016), three of the four comparisons indicate a fold change (TM-scores of 0.46 or lower), and in the remaining case, there is still a large structural change although not necessarily a fold change (TM-score of 0.58). Finally, for the protein from Cabrita et al. (2016), we see the highest TM-scores, meaning the least amount of conformational change, with TM-scores between 0.77 and 0.82.

Note that in this second analysis, at first glance, it might appear that these results show an opposite pattern for the Cabrita et al. (2016) study compared to the other three studies. However, it is just that the results are different (not necessarily opposite), because the data from Cabrita et al. (2016) is quite different than the data from the other three studies. Namely, in the other three studies, the native structure is always a conformation of the last (i.e., sequence-longest) intermediate. This means that in this analysis, for these three studies, we are measuring the 3D structural change over time from a non-native to native intermediate; and there is a major structural change (resulting in a changed overall fold; TM-scores less than or equal to 0.5). On the other hand, in the Cabrita et al. (2016) study, the native structure is the first (rather than last) intermediate, while the conformations of the second (i.e. last) intermediate are non-native structures. This means that for the Cabrita et al. (2016) study, we are measuring the 3D structural change over time from a native to non-native intermediate; and now, there is only a minor change (resulting in a preserved overall fold; TM-scores higher than 0.5). This is because the Cabrita et al. (2016) study investigates a multi-domain

protein composed of an FLN5 and FLN6 domain (Supplementary Section S1.4). Specifically, intermediate 1 corresponds to FLN5 alone, while conformations of intermediates 2a, 2b, and 2c include both an N-terminal FLN5 domain (shown in green) and a C-terminal FLN6 domain (shown in orange) – Supplementary Fig. S2. The consistently high TM-scores (i.e. TM-scores higher than 0.77) observed between green regions across these conformations suggest that FLN5 attains a native-like structure early (i.e., in intermediate 1) and retains a stable structure even after translation of the FLN6 domain. This would explain why the overall fold of the FLN5 domain remains similar despite presence of the FLN6 domain, and thus why the TM-scores remain above 0.5.

Third, in order to evaluate the effect of time passed, we compare conformations corresponding to the same sequence of an intermediate at closer vs. more distant times. Specifically, for the only two studies that allow for this comparison (because they have more than two distinct intermediates in Supplementary Fig. S2) – Hanazono et al. (2018) and Hanazono et al. (2016) – we compare conformational change of the green sequence from time 1 to time 2, and from time 2 to time 3, against its change from time 1 to time 3. For the protein from Hanazono et al. (2018), we find the following. The green sequence in the first intermediate vs. the green sequence in the second intermediate, as well as the green sequence in the second intermediate vs. the green sequence in the third intermediate (i.e. the two pairs of time-closest intermediates), show a high level of conformational changes (TM-scores of 0.43 and 0.42, respectively). Yet, the green sequence in the first intermediate vs. the green sequence in the third intermediate (i.e. the pair of most time-distant intermediates) show an even greater level of conformational change (TM-score of 0.22). Similarly, in Hanazono et al. (2016), the green sequence in the first intermediate vs. the green sequence in the second intermediate, as well as the green sequence in the second intermediate vs. the green sequence in the third intermediate, show some conformational changes (TM-scores of 0.45 and 0.58, respectively). The green sequence in the first intermediate vs. the green sequence in the third intermediate show an even larger conformational change (TM-score of 0.43).

Section S1.8 Additional details on the evaluation strategies used in the Outeiral et al. (2022) study

Here we complement Section 3.2 in the main paper with more details on the evaluation from the study by Outeiral et al. (2022) [46].

Recall that Outeiral et al. (2022) evaluated whether a given method's predicted pathways (i) are predictive of a protein's folding kinetics class (e.g. two-state or multi-state), and (ii) correlate with experimentally measured folding rate constants.

For the first evaluation, the methods' predicted pathways were used to classify whether the given protein folds through two-state or multi-state kinetics, in both an unsupervised and supervised manner. Each predicted classification result was compared to the ground truth data mentioned above. Multiple measures of method performance accuracy were used, including the area under the receiver-operating characteristic (AUROC) curve, which we use as a representative measure for illustration purpose (where AUROC score of 0.50 indicates random performance, and the higher AUROC score, the better the given method). The results (for 10 pathways per method per protein, except for AlphaFold2 with one pathway per protein) are as follows. All methods achieved statistically significant yet quite modest performance, with AUROC scores between 0.56 and 0.675. The actual protein structure prediction methods were compared to a trivial baseline, namely a simple linear classifier based solely on chain length. Surprisingly, this simple baseline outperformed all structure prediction methods, with AUROC score of 0.739. Results were qualitatively similar for the other performance accuracy measures and when predicting 200 pathways per method per protein. A key conclusion from this analysis was that this sequence-agnostic baseline surpassed all structure-based methods in predicting folding kinetics, indicating that the predictive signal captured by current structure prediction tools is weak.

For the second evaluation, the methods were evaluated based on how well their predicted pathways could capture the folding rate constants of proteins that undergo two-state folding kinetics. To test this, Outeiral et al. selected the 79 proteins that had experimentally determined folding rate constants and had at least one pathway classified as two-state. Within these, only the predicted pathways classified as two-state were retained. For each retained pathway, Outeiral et al. determined the relative frame in which folding occurred, defined as the point of maximal increase in native contacts. Outeiral et al. then examined whether this position along the pathway correlated with the experimentally measured folding rate constants. Outeiral et al. compared these correlations of the structure prediction methods to correlations obtained using two baseline methods – average contact order and protein chain length – both being known predictors of folding

rates. The results showed that chain length had the strongest (and correct-sign) correlation, outperforming contact order and any structure prediction method. Most structure prediction methods showed weak or insignificant correlations, and in some cases even the wrong sign. Only AlphaFold2 and another method showed modest, correctly signed correlations, hinting at a limited signal. Outeiral et al.'s findings suggested that while post-translational folding pathways predicted by structure prediction methods may resemble real post-translational pathways in some aspects, they fail to meaningfully correlate with experimental folding rate data.

Section S1.9 Additional details on the methodology used in the Huang et al. (2022) study

Here we complement Section 3.2 in the main paper with more details on the methodology of Pathfinder in the study by Huang et al. (2022) [46].

Pathfinder predicts a protein's post-translational pathway via a three-step framework: seed generation, transition probability exploration, and folding pathway inference.

In the first step, Pathfinder identifies seed states representing key intermediate conformations along a protein's folding pathway. In more detail, Huang et al. (2022) hypothesized that the 3D structures of intermediates that a protein adopts during folding would be captured within, and could thus be extracted from, the ensemble of conformations generated through large-scale simulations. To test this, Huang et al. used Pathfinder to perform large-scale Monte Carlo sampling to produce hundreds of thousands of possible conformations per protein. Since treating every conformation as a seed state was computationally infeasible, clustering was done to group structurally similar conformations. The centroid of each cluster − assumed to be a representative structure − was then selected as a seed state.

In the second step, Pathfinder computes transition probabilities between seed states to map out all the plausible folding pathways a protein might take, based on their likelihood. Because a protein may adopt multiple folding routes, Huang et al. believed that transition probabilities could help identify the most likely sequence of intermediates. In order to compute these transition probabilities, Pathfinder performs a resampling step using a modified energy function that reshapes the energy landscape of a protein − raising deep basins and lowering high barriers − to improve the likelihood of transitions between seed states. Then, structural similarities between seed states are measured using DM-score [63] and compiled into a transition probability matrix. Intuitively, DM-score measures structural similarity by comparing all pairwise residue distances, capturing both local and global structural differences − compared to TM-score which focuses more on global fold differences and is less sensitive to local fold variations.

In the third step, using the transition probability matrix from the previous step, Pathfinder applies dynamic programming to identify the path through the seed states that maximizes the overall transition likelihood. This results in a predicted folding pathway consisting of a small number of key seed states, representing the most probable sequence of structural transitions. The seed states included in this "optimal" pathway are designated as a given protein's predicted post-translational pathway intermediates.

Section S1.10 Additional details on the data from the Pathfinder study by Huang et al. (2022)

Here we complement Section 3.2 in the main paper with more details on the data used in the study by Huang et al. (2022) [46].

Pathfinder was technically applied to 34 proteins in its original study. For 30 of them, some experimental information about their post-translational folding pathways were available from Start2Fold, PFDB, or the literature. The remaining four proteins lacked such data but were included to explore whether Pathfinder could generate new insights into their folding mechanisms. As such, in our paper, we do not discuss results for these four proteins. Instead, we only discuss results for the 30 proteins with some experimental information about their pathways.

Section S1.11 Per-study results of AlphaFold2 predictions of co-translational intermediates

Here we complement Section 3.2.1 in the main paper with *per-study* results related to our analysis of AlphaFold2-predicted co-translational intermediates. Relevant results are shown in Fig. 4 in the main paper.

For the Hanazono et al. (2018) and Hanazono et al. (2016) studies, a common trend in TM-scores is apparent; in each of these studies, AlphaFold2's predicted structure of the last intermediate is highly structurally

similar (with TM-scores greater than 0.80) to the last modeled sequence (i.e. intermediate 3 in Hanazono et al. (2018), and intermediate 4 in Hanazono et al. (2016)). This is because the modeled sequence of the last intermediate in each of these studies is the native structure (as reported in it's respective study). However, most of AlphaFold2's predicted structures of earlier intermediates do not have the same fold as their experimental counterparts (with TM-scores lower than 0.50, with the exception of intermediate 2 in Hanazono et al. (2018) with TM-score = 0.53).

For the Agirrezabala et al. (2022) study a similar trend is also observed, but, with different insights between different conformations of intermediates; for conformation 2c, AlphaFold2's predicted structure is highly structurally similar to it (with TM-score = 0.95), because the modeled sequence of this conformation is the native structure. In contrast, for conformation 2a – an experimentally observed non-native structure – AlphaFold2's predicted structure is highly dissimilar to it, close to random (with TM-score = 0.22). Although these two conformations have the same deposited sequences, when given a modeled sequence as input, AlphaFold2's predicted structure is biased towards predicting a native structure with high confidence, rather than a structure of an intermediate. Furthermore, Alphafold2 cannot correctly predict any of the first intermediate conformations (i.e. conformations 1a, 1b, and 1c, with TM-scores less than or equal to 0.17).

At first glance, for the Cabrita et al. (2016) study, results show a different pattern, with AlphaFold2's predicted structure of the first intermediate being highly structurally similar to the modeled sequence of the first intermediate (TM-score = 0.82), and AlphaFold2's predicted structures of the conformations of the second intermediate not having the same fold for most of the conformations (with TM-scores ranging between 0.45 and 0.53). However, the first intermediate is the native structure (as reported in the Cabrita et al. study), and the conformations of the second intermediate are non-native structures. While AlphaFold2 accurately predicts the native structure (i.e. intermediate 1), it is unable to predict any conformations of the second intermediate with high structural similarity to experimental data.

Section S1.12 Additional details on structural similarity between experimentally determined intermediates and their corresponding "proxy" vs. AlphaFold2-predicted intermediates

Here we complement Section 3.2.2 in the main paper with more details about the analysis of 3D structural similarity between experimentally determined intermediates and their corresponding "proxy" vs. AlphaFold2-predicted intermediates. Relevant results are shown in Supplementary Table S1.

In this analysis, of the four studies/proteins from Supplementary Fig. S2, we focus on those studies that meet two key criteria. First, for a given protein, its native structure must correspond to the intermediate with the longest sequence out of all intermediates. This ensures that the native structure corresponds to the full-length conformation, which is necessary for generating "proxy" intermediates by extracting substructures from the native fold. The protein from the Cabrita et al. (2016) study is excluded from further analysis because it violates this criterion: none of the three conformations of the intermediate with the longest sequence (conformations a, b, and c of intermediate 2) are the native structure; the native structure corresponds to the shortest-sequence intermediate (intermediate 1) (Supplementary Fig. S2). Second, for a given protein, only a single conformation should be available for any intermediate. This constraint is desirable because if multiple different conformations exist for the same sequence, AlphaFold2 would incorrectly predict the same 3D structure for all of those confirmations, given their shared sequence. The protein from the Agirrezabala et al. (2022) study fails to meet this criterion, as it has three conformations for its intermediate 1 and two conformations for its intermediate 2; also, the protein from the Cabrita et al. (2016) study fails this criterion too, as it has three confirmations for its intermediate 2 (Supplementary Fig. S2). Only the proteins from the Hanazono et al. (2018) and Hanazono et al. (2016) studies satisfy both conditions and are thus considered in the rest of this analysis.

We infer "proxy" intermediates for the proteins in these two studies as follows: For the protein in the Hanazono et al. (2018) study, we use the native structure (i.e. the experimentally determined 3D structure of intermediate 3) to extract the 3D substructure corresponding to the experimentally determined sequence of intermediate 1 from Supplementary Fig. S2. This substructure is the "proxy" for intermediate 1. Similarly, we use the same native structure to extract the substructure corresponding to the experimentally determined sequence of intermediate 2 from Supplementary Fig. S2. This substructure is the "proxy" for intermediate 2. For the protein in the Hanazono et al. (2016) study, we follow the same procedure using the native structure (i.e. the experimentally determined 3D structure of intermediate 4) to generate "proxy" intermediates for the experimentally determined sequences corresponding to intermediates 1 and 2 from Supplementary Fig. S2. Note that we do not construct "proxy" intermediates for the native structures themselves, as this would yield

structurally identical models by definition, resulting in TM-scores of 1.0 and creating an unfair comparison to AlphaFold2's predictions – which are not guaranteed to yield perfect structural similarity to experimentally determined structures. In total, two "proxy" intermediates are constructed for each protein in each of the two considered studies, and their structural similarities to their respective experimentally determined counterparts are computed using TM-score (these scores were originally computed in Supplementary Fig. S2, but are shown again for the purpose of this analysis in Supplementary Table S1).

We infer AlphaFold2-predicted intermediates for the proteins in the two considered studies as follows: For the protein in the Hanazono et al. (2018) study, we input the experimentally determined sequence of intermediate 1 from Supplementary Fig. S2 into AlphaFold2 to obtain the corresponding AlphaFold2-predicted intermediate; similarly, we input the experimentally determined sequence of intermediate 2 from Supplementary Fig. S2 into AlphaFold2 to obtain the corresponding AlphaFold2-predicted intermediate. For the protein in the Hanazono et al. (2016) study, we follow the same procedure using the experimentally determined sequences of intermediates 1 and 2 from Supplementary Fig. S2 as inputs into AlphaFold2 to obtain their corresponding AlphaFold2-predicted intermediates. Note that we do not generate AlphaFold2-predicted intermediates for the native structures themselves for the same reason as stated above. In total, two AlphaFold2-predicted intermediates are generated for each protein from each of the two considered studies, and their structural similarities to their respective experimentally determined counterparts are computed using TM-score (these scores were originally computed in Fig. 4 in the main paper, but are shown again for the purpose of this analysis in Supplementary Table S1).

Section S2 Supporting figures and tables for the main paper

Study	Protein name	PDB ID	Intermediate	Deposited sequence range (starting - ending)	Modeled sequence range (starting - ending)	TM-score
**Neudecker, P., et al.** (2012)	Fyn SH3	2L2P	***1*** 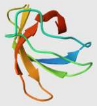	(1 - 66) (early stage)	**(1 - 56)** (early stage)	0.80
		2LP5	***2*** 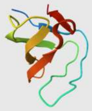	(1 - 66) (final stage)	**(1 - 59)** (final stage)	
**Zhou, Z., et al.** (2008)	Ribonuclease H	2RPI	***1*** 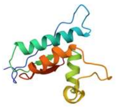	(1 - 112) (early stage)	**(1 - 106)** (early stage)	0.77
		1RIL	***2*** 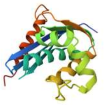	(1 - 166) (final stage)	**(1 - 147)** (final stage)	

Figure S1: Details of the two studies we have identified that report 3D structural data of post-translational intermediates. For each study, i.e. its considered protein, two PDB IDs and two visualizations are shown for the protein's two intermediates (1: "early-stage" or pre-native state; 2: "final stage" or native state). In the fourth and fifth columns, the deposited sequence range and the modeled sequence range for each intermediate is provided, as reported in its respective study and PDB. Note that a bolded model sequence range value indicates that there is a range discrepancy between the reported deposited sequence and the reported model sequence. In the last column, we measure and show the structural similarity between the modeled sequence of the first intermediate and the modeled sequence of the second intermediate using TM-score [62].

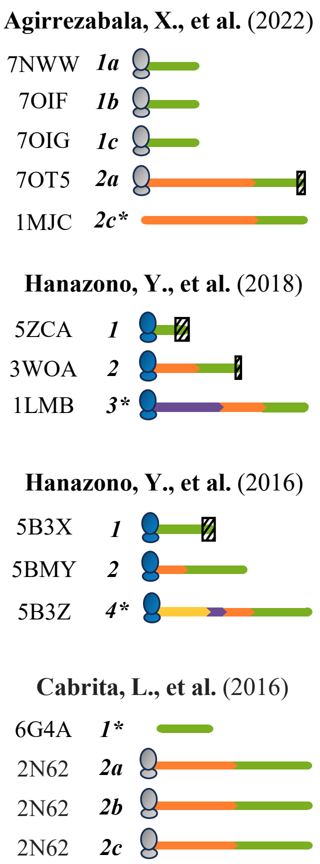

Agirrezabala, X., et al. (2022)

Compared conformations	*TM-score*
1a – 1b	0.13
1a – 1c	0.26
1b – 1c	0.29
1a – 2a (green only)	0.33
1a – 2c* (green only)	0.16
1b – 2a (green only)	0.14
1b – 2c* (green only)	0.15
1c – 2a (green only)	0.16
1c – 2c* (green only)	0.14
2a (green only) – 2c* (green only)	0.28
2a – 2c*	0.22

Hanazono, Y., et al. (2018)

Compared conformations	*TM-score*
1 – 2 (green only)	0.43
1 – 3* (green only)	0.22
2 (green only) – 3* (green only)	0.42
2 – 3* (green+orange only)	0.27

Hanazono, Y., et al. (2016)

Compared conformations	*TM-score*
1 – 2 (green only)	0.45
1 – 4* (green only)	0.43
2 (green only) – 4* (green only)	**0.58**
2 – 4* (green+orange only)	0.46

Cabrita, L., et al. (2016)

Compared conformations	*TM-score*
1* – 2a (green only)	**0.82**
1* – 2b (green only)	**0.80**
1* – 2c (green only)	**0.77**
2a (green only) – 2b (green only)	**0.80**
2a (green only) – 2c(green only)	**0.77**
2b (green only) – 2c (green only)	**0.82**
2a – 2b	0.49
2a – 2c	0.47
2b – 2c	0.44

Figure S2: Structural similarities in terms of TM-score between different combinations of (the conformations of) the co-translational intermediates from the same protein/study. The four proteins/studies considered are the same as in Fig. 3 in the main paper. Their conformations of the intermediates are shown again on the left side of the current figure for easiness, with the exception of the conformations with PDB IDs 7OII and 5B3Y, which are excluded from any analyses, per the discussion in Fig. 3 in the main paper; all colors and hashed regions in the current figure match those in Fig. 3 in the main paper. On the right side of the figure are four tables corresponding to the four proteins/studies. In each table, TM-scores are shown between all possible pairs of conformations of the intermediates when accounting for their modeled sequence regions. All TM-scores with values higher than 0.50 are bolded, corresponding to structures that have the same overall fold [62].

Agirrezabala, X., et al. (2022)

	PDB ID	Intermediate	Modeled sequence range (starting - ending)	TM-score *Rank 1*	*Rank 2*	*Rank 3*	*Rank 4*	*Rank 5*	Max TM-score difference
1a	7NWW		(1-27)	0.15	0.14	0.14	0.14	0.14	0.01
1b	7OIF		(1-27)	0.14	0.15	0.17	0.15	0.15	0.03
1c	7OIG		(1-27)	0.17	0.15	0.15	0.15	0.15	0.02
2a	7OT5		**(1-66)**	0.22	0.22	0.22	0.22	0.22	0.00
2c*	1MJC		(1-70)	**0.95**	**0.95**	**0.95**	**0.95**	**0.95**	0.00

Hanazono, Y., et al. (2018)

	PDB ID	Intermediate	Modeled sequence range (starting - ending)	TM-score *Rank 1*	*Rank 2*	*Rank 3*	*Rank 4*	*Rank 5*	Max TM-score difference
1	5ZCA		**(1-12)**	0.41	0.42	0.43	0.42	0.45	0.04
2	3WOA		**(1-42)**	**0.53**	**0.50**	**0.57**	**0.51**	0.48	0.09
3*	1LMB		(1-92)	**0.96**	**0.95**	**0.96**	**0.96**	**0.95**	0.01

Hanazono, Y., et al. (2016)

	PDB ID	Intermediate	Modeled sequence range (starting - ending)	TM-score *Rank 1*	*Rank 2*	*Rank 3*	*Rank 4*	*Rank 5*	Max TM-score difference
1	5B3X		**(1-8)**	0.28	0.21	0.33	0.28	0.30	0.12
2	5BMY		(1-17)	0.21	0.21	0.26	0.30	0.26	0.09
4*	5B3Z		(1-35)	**0.80**	**0.81**	**0.82**	**0.77**	**0.84**	0.07

Cabrita, L., et al. (2016)

	PDB ID	Intermediate	Modeled sequence range (starting - ending)	TM-score *Rank 1*	*Rank 2*	*Rank 3*	*Rank 4*	*Rank 5*	Max TM-score difference
1*	6G4A		(1-114)	**0.82**	**0.81**	**0.81**	**0.81**	**0.80**	0.02
2a	2N62		(1-221)	0.47	**0.51**	**0.51**	**0.51**	**0.52**	0.05
2b	2N62		(1-221)	**0.53**	**0.56**	**0.55**	**0.58**	**0.56**	0.05
2c	2N62		(1-221)	0.45	0.44	0.44	0.44	0.44	0.01

Figure S3: The same type of results as in Fig. 4 in the main paper, except that we report TM-scores for all five top-ranked AlphaFold2-predicted structures. Specifically, the figure shows structural similarities in terms of TM-scores between Alphafold2-predicted structures and experimentally determined structures, for the same 15 conformations of the 10 co-translational intermediates as in Supplementary Fig. S2, i.e. for all but the two red ones in Fig. 3 in the main paper. For each of the four studies, there is a corresponding table; the first three table columns are already explained in Fig. 3 in the main paper and Supplementary Fig. S2 (except that here we do not show or analyze an attached entity at the C-terminus). The fourth table column reports the TM-scores of all five highest-ranked predicted structures from AlphaFold2. All TM-scores with values higher than 0.50 are bolded, corresponding to structures that have the same overall fold. The fifth (last) column reports the maximum absolute TM-score difference between all pairs of the five AlphaFold2-predicted structures for a given (conformation of the) intermediate.

Agirrezabala, X., et al. (2022)

	PDB ID	Intermediate	Modeled sequence range (starting - ending)	TM-score
1a	7NWW		(1-27)	0.15
1b	7OIF		(1-27)	0.14
1c	7OIG		(1-27)	0.17
2a	7OT5		**(1-66)**	0.22
*2c**	1MJC		(1-70)	**0.95**

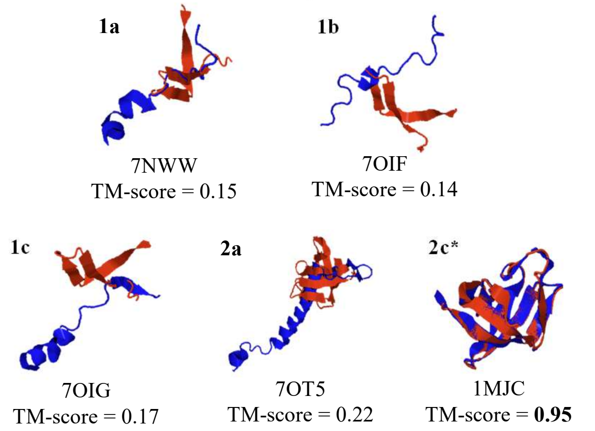

Hanazono, Y., et al. (2018)

	PDB ID	Intermediate	Modeled sequence range (starting - ending)	TM-score
1	5ZCA		**(1-12)**	0.41
2	3WOA		**(1-42)**	**0.53**
*3**	1LMB		(1-92)	**0.96**

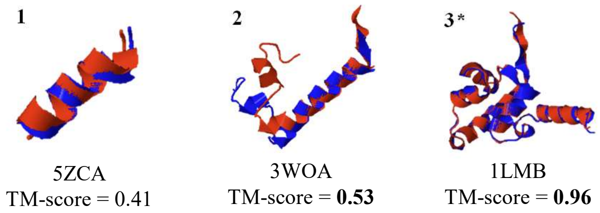

Hanazono, Y., et al. (2016)

	PDB ID	Intermediate	Modeled sequence range (starting - ending)	TM-score
1	5B3X		**(1-8)**	0.28
2	5BMY		(1-17)	0.21
*4**	5B3Z		(1-35)	**0.80**

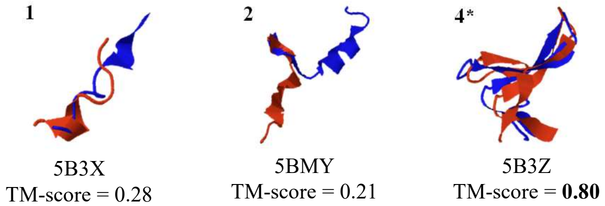

Cabrita, L., et al. (2016)

	PDB ID	Intermediate	Modeled sequence range (starting - ending)	TM-score
*1**	6G4A		(1-114)	**0.82**
2a	2N62		(1-221)	0.47
2b	2N62		(1-221)	**0.53**
2c	2N62		(1-221)	0.45

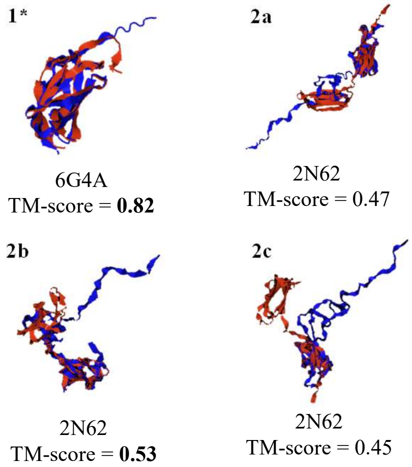

Figure S4: Structural similarities in terms of TM-scores between Alphafold2-predicted structures and experimentally determined structures for the same 15 conformations of the 10 co-translational intermediates as in Supplementary Fig. S2, i.e. for all but the two red ones in Fig. 3 in the main paper. For each of the four studies, there is a corresponding table; the first three table columns are already explained in Fig. 3 in the main paper and Supplementary Fig. S2 (except that here we do not show or analyze an attached entity at the C-terminus). The fourth table column reports the TM-score of the highest-ranked predicted structure from AlphaFold2 (i.e. rank 1 from Supplementary Fig. S3). All TM-scores with values higher than 0.50 are bolded, corresponding to structures that have the same overall fold. For each TM-score, corresponding to a conformation of an intermediate, a 3D visualization of the AlphaFold2 predicted structure (blue) is overlaid with a 3D visualization of the experimental 3D structure corresponding to the modeled sequence (red).

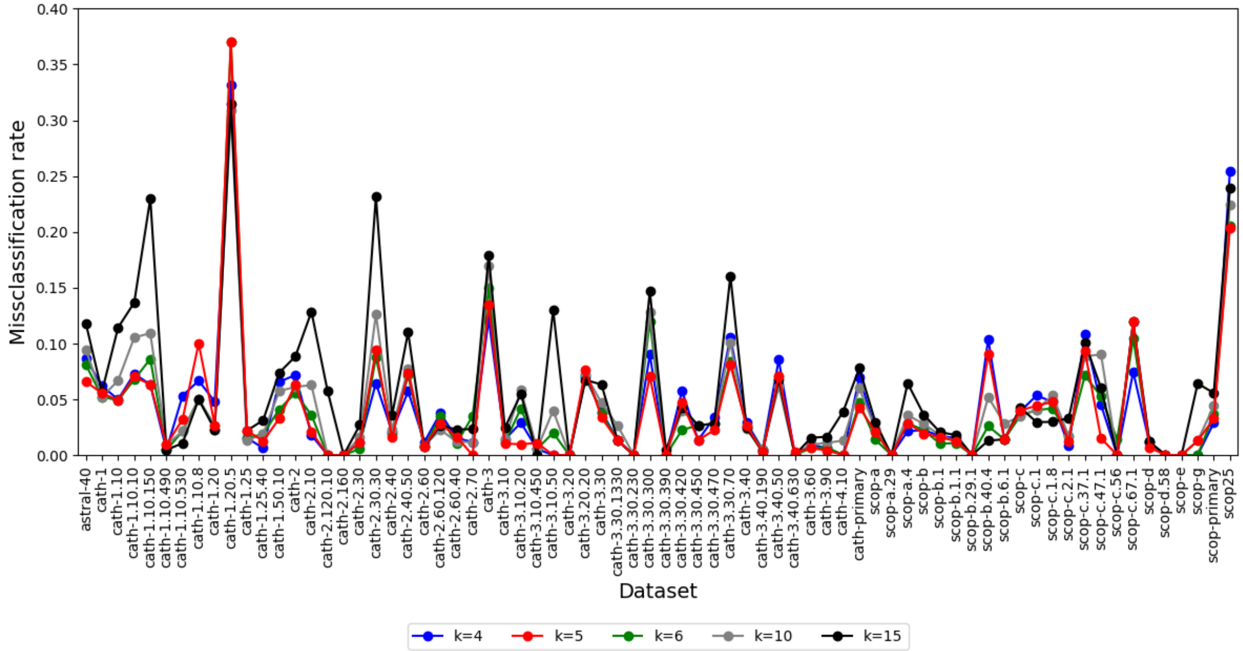

Figure S5: The effect of constructing dynamic PSNs with alternative values for k. Recall from Section 3.2.2 in the main paper that dynamic PSNs were evaluated in the PSC task of classifying ∼44,000 protein domains with respect to their CATH [19] and SCOPe [13] structural classes. In the original study [41], the ∼44,000 protein domains were organized into 72 protein domain datasets (x-axis). Also in the original study, dynamic PSNs constructed when $k = 5$ (red in the figure; Section 3.2.2 in the main paper) were evaluated in the task of PSC with respect to misclassification rate (y-axis, where lower is better). As an original contribution to our current study, we evaluate the effect on PSC misclassification rate of constructing dynamic PSNs with alternative values for k (specifically $k = 4, 6, 10, 15$, corresponding to blue, green, grey, and black in the figure, respectively). We find that out of the 72 datasets, $k = 5$ performs the best (i.e. has the lowest misclassification rate) on 34 datasets, and is within 1% misclassification rate of the best other value(s) of k on an additional 24 datasets. In other words, it is (close to) tied with, or better than, all other considered values of k on $(34 + 24)/72 = 80.6\%$ of all analyzed datasets. These findings confirm that $k = 5$ is quite a meaningful parameter choice for constructing dynamic PSNs.

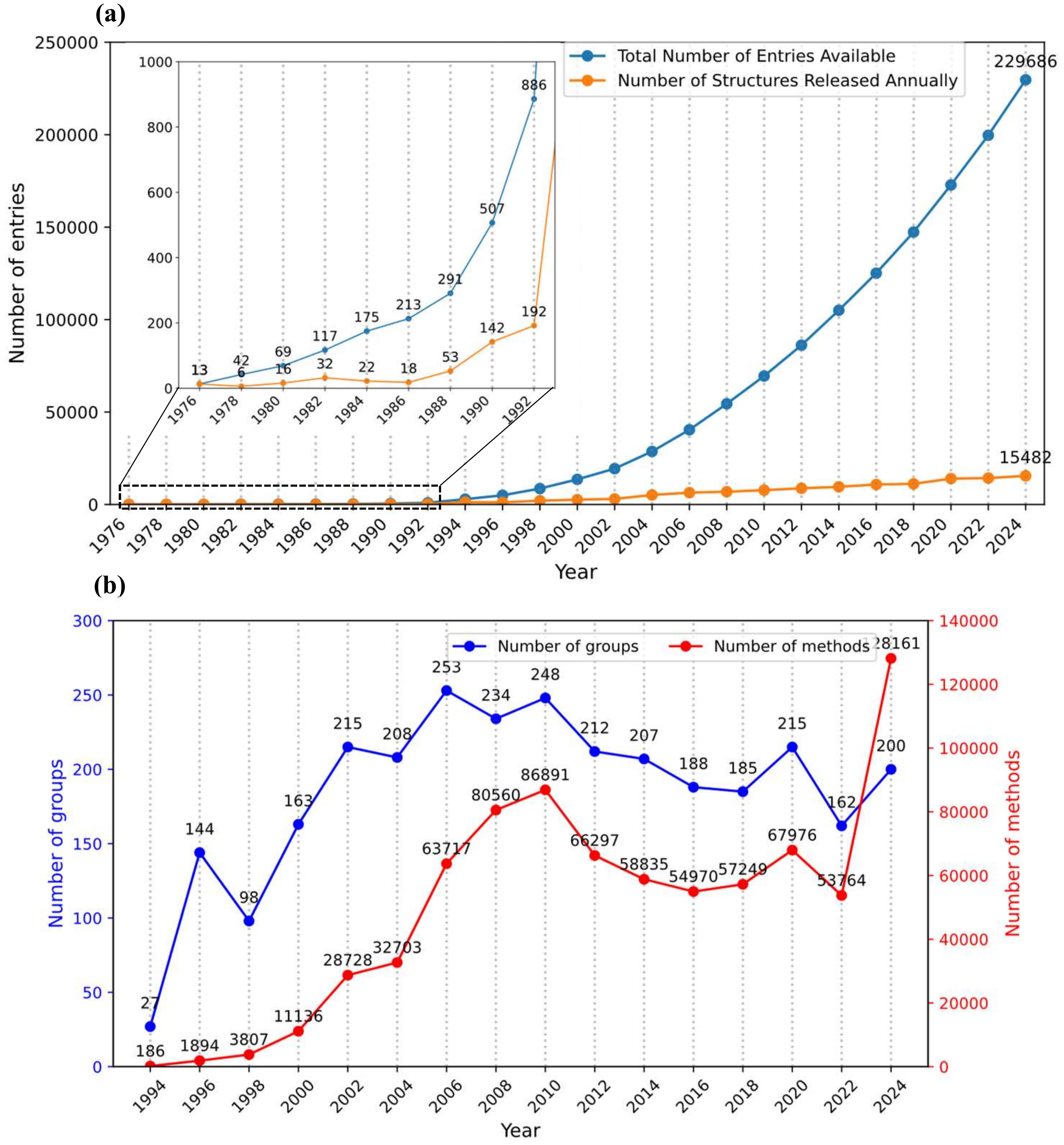

Figure S6: The growth of **(a)** available protein 3D structures (i.e. entries) in PDB over time and **(b)** participating research groups and submitted computational predictive methods (also referred to as "models" in some literature) in the CASP competition over time. We collected these statistics from `https://www.rcsb.org/stats` and `https://predictioncenter.org/index.cgi`, respectively.

Study	*PDB ID*	*Exp. intermediate*	*TM-score of "proxy" intermediate vs. exp. intermediate*	*TM-score of AlphaFold2 intermediate vs. exp. intermediate*
**Hanazono, Y., et al.** (2018)	5ZCA	1	0.22	0.41
	3WOA	2	0.27	**0.53**
**Hanazono, Y., et al.** (2016)	5B3X	1	0.43	0.28
	5BMY	2	0.46	0.21

Table S1: Results on whether experimentally (exp.) determined intermediates are structurally matched better or worse by their corresponding "proxy" intermediates or by their corresponding AlphaFold2-predicted intermediates. For reasons stated in the text, this analysis is done on two of the four studies/proteins from Supplementary Fig. S2, specifically Hanazono et al. (2018) [21] and Hanazono et al. (2016) [20], corresponding to the two sub-tables in the table. In each sub-table, the two PDB ID's corresponding to the two *non-native* intermediates of a protein are shown in the first and second rows, respectively. For each intermediate, two TM-scores are shown, one for "proxy" intermediate vs. experimental intermediate, and one for AlphaFold2-predicted intermediate vs. experimental intermediate. All TM-scores with values higher than 0.50 are bolded, corresponding to structures that have the same overall fold.
